# Fluticasone Propionate Suppresses Poly(I:C)-Induced ACE2 in Primary Human Nasal Epithelial Cells

**DOI:** 10.3389/fcimb.2021.655666

**Published:** 2021-04-26

**Authors:** Akira Nakazono, Yuji Nakamaru, Mahnaz Ramezanpour, Takeshi Kondo, Masashi Watanabe, Shigetsugu Hatakeyama, Shogo Kimura, Aya Honma, P. J. Wormald, Sarah Vreugde, Masanobu Suzuki, Akihiro Homma

**Affiliations:** ^1^ Department of Otolaryngology-Head and Neck Surgery, Faculty of Medicine and Graduate School of Medicine, Hokkaido University, Sapporo, Japan; ^2^ Department of Surgery–Otorhinolaryngology Head and Neck Surgery, Central Adelaide Local Health Network and the University of Adelaide, Adelaide, SA, Australia; ^3^ Department of Biochemistry, Faculty of Medicine and Graduate School of Medicine, Hokkaido University, Sapporo, Japan

**Keywords:** SARS-CoV-2, TMPRSS2, virus infection, toll-like receptors, intranasal steroid spray, NFκB, interferon, COVID-19

## Abstract

**Background:**

From the first detection in 2019, SARS-CoV-2 infections have spread rapidly worldwide and have been proven to cause an urgent and important health problem. SARS-CoV-2 cell entry depends on two proteins present on the surface of host cells, angiotensin-converting enzyme 2 (ACE2) and transmembrane protease serine 2 (TMPRSS2). The nasal cavity is thought to be one of the initial sites of infection and a possible reservoir for dissemination within and between individuals. However, it is not known how the expression of these genes is regulated in the nasal mucosa.

**Objective:**

In this study, we examined whether the expression of ACE2 and TMPRSS2 is affected by innate immune signals in the nasal mucosa. We also investigated how fluticasone propionate (FP), a corticosteroid used as an intranasal steroid spray, affects the gene expression.

**Methods:**

Primary human nasal epithelial cells (HNECs) were collected from the nasal mucosa and incubated with Toll-like receptor (TLR) agonists and/or fluticasone propionate (FP), followed by quantitative PCR, immunofluorescence, and immunoblot analyses.

**Results:**

Among the TLR agonists, the TLR3 agonist Poly(I:C) significantly increased ACE2 and TMPRSS2 mRNA expression in HNECs (ACE2 36.212±11.600-fold change, p<0.0001; TMPRSS2 5.598±2.434-fold change, p=0.031). The ACE2 protein level was also increased with Poly(I:C) stimulation (2.884±0.505-fold change, p=0.003). The Poly(I:C)-induced ACE2 expression was suppressed by co-incubation with FP (0.405±0.312-fold change, p=0.044).

**Conclusion:**

The activation of innate immune signals *via* TLR3 promotes the expression of genes related to SARS-CoV2 cell entry in the nasal mucosa, although this expression is suppressed in the presence of FP. Further studies are required to evaluate whether FP suppresses SARS-CoV-2 viral cell entry.

## Introduction

Corona virus disease 2019 (COVID-19) has rapidly spread to become the most urgent health issue around the world. Severe acute respiratory syndrome coronavirus 2 (SARS-CoV-2) has been identified as the responsible virus for COVID-19. SARS-CoV-2 can infect multiple organs, including the intestines, esophagus, liver, heart, kidneys, bladder, testes, brain, lung and nasal cavity (Chen et al., 2020; Huang et al., 2020; Zhang et al., 2020; Zou et al., 2020).

Among these organs, the nasal cavity is assumed to be one of the organs most susceptible to SARS-CoV-2 infection (Hou et al., 2020). It has been reported that more than half of COVID-19 patients had nasal symptoms including olfactory dysfunction and nasal obstruction (Paderno et al., 2020; Sakalli et al., 2020). A high SARS-CoV-2 viral load was indeed detected in nasal swabs from COVID-19 patients (Zou et al., 2020). The nasal cavity is also considered to be the initial site of the infection and is thought to act as a possible reservoir for dissemination within and between individuals (Hou et al., 2020; Sungnak et al., 2020). Therefore, it is important to elucidate the mechanism of SARS-CoV-2 infection in the nasal mucosa, not only to evaluate how it affects local inflammation in the nasal cavity but also to understand whether topical therapies might affect infection efficiency and potentially reduce the development of systemic infection and transmission to other individuals.

For its entry into host cells, SARS-CoV-2 depends on angiotensin-converting enzyme 2 (ACE2) and transmembrane protease serine 2 (TMPRSS2) expressed on the surface of the cells (Li et al., 2003; Hoffmann et al., 2020). SARS-CoV-2 attaches to the cell membrane using spike (S) proteins to bind to ACE2 on the host cells. The viral cell entry also requires S protein-priming by TMPRSS2 (Hoffmann et al., 2020). Therefore, the expression of ACE2 and TMPRSS2 is assumed to be associated with host susceptibility to and severity of COVID-19 (Bunyavanich et al., 2020; Devaux et al., 2020; Saheb Sharif-Askari et al., 2020; Sharif-Askari et al., 2020; Wu et al., 2020). Notably, differences in ACE2 and TMPRSS2 expression levels in the lower airway might contribute to COVID-19 severity (Matsumoto and Saito, 2020; Saheb Sharif-Askari et al., 2020). In the nasal epithelium, ACE2 and TMPRSS2 have been shown to be expressed at high levels (Sungnak et al., 2020; Ziegler et al., 2020), and their expression has been related to the susceptibility of the nasal cavity mucosa to SARS-CoV2 infection (Bunyavanich et al., 2020; Sharif-Askari et al., 2020). These proteins have also been targeted as a possible therapeutic strategy for COVID-19 (Hoffmann et al., 2020; Patel and Verma, 2020; Wu et al., 2020).

Recent research has shown that ACE2 expression is induced by interferons (IFNs), suggesting that ACE2 expression could be regulated by the activation of innate immune signals such as Toll-like receptor (TLR) signals (Ziegler et al., 2020). Also, corticosteroids, which are a regulator of innate immunity (Kato et al., 2007; Zhang et al., 2007), might affect ACE2 expression. However, it is not known whether the activation or suppression of innate immune signaling affects ACE2 expression in the nasal mucosa.

In this study, we examined whether TLR agonist activation alters ACE2 and TMPRSS2 expression levels in primary human nasal epithelial cells (HNECs). We also investigated whether fluticasone propionate (FP), a corticosteroid widely used as intranasal steroid spray, affects the expression of SARS-CoV-2 cell entry-related genes.

## Materials and Methods

### Primary Human Nasal Epithelial Cell Cultures

This study was approved by the Institutional Review Board for Clinical Research of Hokkaido University Hospital, Sapporo, Japan (019–0242) and by The Queen Elizabeth Hospital Human Research Ethics Committee (reference HREC/15/TQEH/132), and was conducted in accordance with the Declaration of Helsinki. Written informed consent was obtained from study participants prior to tissue or cell collection. Primary human nasal epithelial cells (HNECs) were taken from the nasal mucosa of the inferior turbinates of seven patients treated for deviation of the nasal septum as previously described (Cooksley et al., 2015; Suzuki et al., 2018; Ramezanpour et al., 2019). The cells were suspended in Bronchial Epithelial Growth Medium (BEGM, CC-3170, Lonza, Walkersville, MD, USA) supplemented with Amphotericin B (Fujifilm, Osaka, Japan) and Penicillin/Streptomycin (Fujifilm, Osaka, Japan). Monocytes were depleted from the cell suspension using anti-CD68 antibody (Dako, Glostrup, Denmark) -coated cell culture dishes. HNECs were incubated at 37°C in humidified conditions under 5% CO_2_ in collagen-coated flasks (Thermo Scientific, Waltham, MA, USA) until passage 2.

### TLR Stimulation and Agents

HNECs were seeded onto 6-well dishes coated with collagen type I (Kurabo, Osaka, Japan) at 0.6 × 10^6^ cells/well for 24 hours prior to subsequent analysis. The cells were washed with phosphate-buffered saline (PBS), followed by the addition of TLR agonists (Invivogen, San Diego, CA, USA) for 24 hours, unless otherwise described. The following dosages were used: TLR1/2 (Pam3CSK4) 1 µg/ml, TLR2 (HKLM) 10^8^ cells/ml, TLR3 (Poly(I:C)) 10 µg/ml, TLR4 (LPS) 10 µg/ml, TLR5 (Flagellin) 10 µg/ml, TLR6/2 (FSL-1) 1 µg/ml, TLR7 (Imiquimod) 10 µg/ml, TLR8 (ssRNA40) 10 µg/ml, and TLR9 (ODN2006) 5 µM. In some experiments, fluticasone propionate (FP; Cayman Chemical Company, Ann Arbor, MI, USA) and JSH-23 (NFκB transcriptional activity inhibitor; Abcam, Cambridge, MA, USA), was added to the cells, 1 hour prior to TLR stimulation. The following dosages were used: FP 10 nM and JSH-23 30 µM, unless otherwise described. FP and JSH-23 were dissolved in dimethyl sulfoxide (DMSO). In experiments where FP or JSH-23 was used, corresponding DMSO control solutions were also used in parallel as controls.

### RNA Extraction, Reverse Transcription, and qPCR

HNECs were incubated in 6-well dishes in BEGM media. After incubation with the TLR agonists, FP and/or JSH-23, HNECs were collected and washed with PBS, followed by RNA extraction using ISOGEN in accordance with the manufacturer’s instructions (Nippon Gene, Tokyo, Japan). Extracted RNA was quantified using a Nanodrop 1000 spectrophotometer (Thermo Fisher Scientific, Waltham, MA, USA). RNA was reverse transcribed into cDNA using a ReverTra Ace qPCR RT Kit (TOYOBO, Osaka, Japan) with a LifeECO Thermal Cycler (Hangzhou Bioer Technology, Hangzhou, China). The resulting cDNA was subjected to qPCR using a StepOne Realtime PCR System (Applied Biosytems, Foster City, CA, USA) with Power SYBR Green PCR Master Mix (Thermo Fisher Scientific, Waltham, MA, USA) and nuclease-free water (Thermo Fisher Scientific, Waltham, MA, USA). The average threshold cycle (Ct) was determined from three or more independent experiments and the level of gene expression relative to glyceraldehyde 3-phosphate dehydrogenase (GAPDH) was determined using the comparative CT method. The following primers were used for the qPCR: GAPDH forward (Fw) 5’-TGCACCACCAACTGCTTAGC-3’, GAPDH reverse (Rev) 5’-GGCATGGACTGTGGTCATGAG-3’, ACE2 (Fw) 5’-CTCTACAGAAGCTGGACAGAAAC-3’, ACE2 (Rev) 5’-GAGCAGTGGCCTTACATTCA-3’, TMPRSS2 (Fw) 5’-GGAGTGTACGGGAATGTGATG-3’, TMPRSS2 (Rev) 5’-GGACGAAGACCATGTGGATTAG-3’, TNF-α (Fw) 5’-GAGGCCAAGCCCTGGTATG-3’, TNF-α (Rev) 5’-CGGGCCGATTGATCTCAGC-3’, IL-6 (Fw) 5’-ATGTAGCCGCCCCACACAGA-3’, IL-6 (Rev) 5’-ATTTGCCGAAGAGCCCCTCAG-3’, IFN-β (Fw) 5’-AGGACAGGATGAACTTTGAC-3’, IFN-β (Rev) 5’-TGATAGACATTAGCCAGGAG-3’, CXCL10 (Fw) 5’-GCTCTACTGAGGTGCTATGTTC-3’, CXCL10 (Rev) 5’-GGAGGATGGCAGTGGAAGTC-3’, IFN-*γ* (Fw) 5’-TCGGTAACTGACTTGAATGTCCA-3’, and IFN-*γ* (Rev) 5’-TCGCTTCCCTGTTTTAGCTGC-3’.

### Immunoblotting

HNECs were incubated with 10 µg/ml of Poly(I:C), 10 nM of FP for 28 hours. The cells were lysed in glycoprotein denaturing buffer containing sodium dodecyl sulfate (0.5% (w/v)) and 40 mM dithiothreitol. The cell lysates were then boiled and sonicated in glycoprotein denaturing buffer. PNGase F (New England Biolabs, Ipswich, MA, USA) was used to remove oligosaccharides from the glycoproteins following the manufacturer’s protocol. After removing oligosaccharides, SDS sample buffer containing 50 mM Tris-HCl (pH 6.8), 2-mercaptoethanol (6% (v/v)), sodium dodecyl sulfate (2% (w/v)), glycerol (10% (v/v)) and bromophenol blue (0.01% (w/v)) was added to the supernatant. Immunoblot analysis was performed with the antibodies indicated below. Immune complexes were detected with horseradish peroxidase-conjugated antibodies to rabbit (1:10000 dilution, Promega, Madison, WI, USA) and an enhanced chemiluminescence system (Thermo Fisher Scientific, Waltham, MA, USA). The acquired bands were quantified using ImageJ 1.50i (National Institutes of Health, Bethesda, MD, USA) and expressed as the ratio relative to GAPDH. The antibodies used in immunoblotting were as follows: rabbit monoclonal anti-ACE2 (1:1000; SN0754, Thermo Fisher Scientific, Waltham, MA, USA), rabbit monoclonal anti-TMPRSS2 (1:1000; ab92323, Abcam, Cambridge, United Kingdom), and monoclonal anti-GAPDH HRP-DirecT (1:5000; M171-7, MBL, Nagoya, Japan).

### Immunofluorescence

HNECs were incubated for 48 hours with or without 10 µg/ml Poly(I:C) and 10 nM fluticasone propionate (FP). HNECs were fixed with 2.5% formalin in PBS for 10 minutes at room temperature (RT) followed by washing twice with PBS. Fixed samples were blocked for 1 hour with Protein Block (SFB; Dako, Glostrup, Denmark). Rabbit anti-ACE2 polyclonal antibody (1:100, Invitrogen, Carlsbad, CA, USA) and rabbit anti-TMPRSS2 antibody (1:100, Abcam, Cambridge, MA, USA) were added overnight at 4°C. Excess primary antibody was removed, and 2 μg/ml anti-mouse Alexa-Fluor 488 conjugated secondary antibody (Jackson ImmunoResearch Labs Inc., West Grove, PA, USA) was added and incubated for 1 hour at RT. The samples were rinsed in TBST, and after the third wash, 200 ng/ml of 4′, 6-diamidino-2-phenylindole (DAPI; Sigma, Aldrich, USA) was added to resolve nuclei. Samples were visualized using a LSM700 confocal laser scanning microscope (Zeiss Microscopy, Oberkochen, Germany). Processing was performed using ZEN Imaging Software (Carl Zeiss AG, Oberkochen, Germany). The threshold of each image was adjusted for each channel to remove background fluorescence and evaluation was performed in a blinded fashion. ACE2 and TMPRSS2 fluorescence intensities were quantified and normalized to the DAPI intensity. Results are expressed as the relative value of mean arbitrary fluorescence units as provided by the ZEN imaging software.

### Statistical Analysis

All data was expressed as mean ± standard deviation (SD). Shapiro-Wilk test and Kolmogrov test were applied to examine whether the data fitted normal and lognormal distributions, respectively. In cases where the data fitted the lognormal distribution, log transformation was performed before statistical analysis. Fold changes in relative gene expression were compared by a two-tailed t-test. When 3 or more groups were compared, one-way Analysis of Variance (ANOVA) followed by Dunnetts’s test was used to analyze differences among the groups. P values of <0.05 were considered statistically significant. All analyses were performed using JMP^®^ 11 software (SAS Institute Inc., Cary, NC, USA).

## Results

### Poly(I:C) Increases ACE2 and TMPRSS2 mRNA Expression Levels

**Figure 1:**
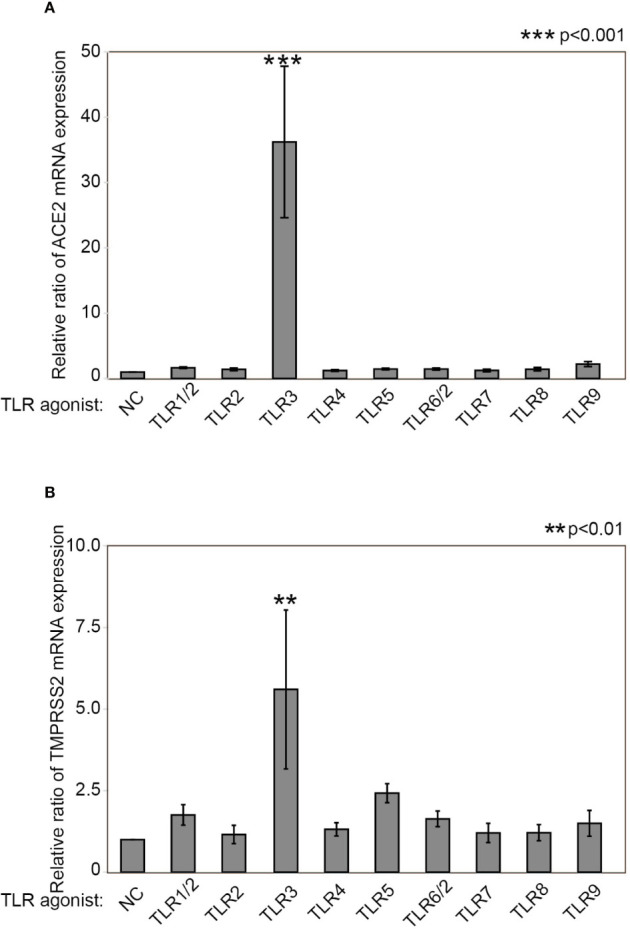
Poly(I:C) increases ACE2 mRNA as well as TMPRSS2 expression. Relative mRNA expression of ACE2 **(A)** and TMPRSS2 **(B)** after stimulation with TLR agonists for 24 hours. The following TLR agonists were used: Pam3CSK4 for TLR1/2, HKLM for TLR2, Poly(I:C) for TLR3, LPS for TLR4, Flagellin for TLR5, FSL-1 for TLR6/2, Imiquimod for TLR7, ssRNA40 for TLR8, and ODN2006 for TLR9. Relative mRNA expression was determined by normalization against untreated control cells and GAPDH. Data are means ± standard deviation (s.d.) of values from three independent experiments. **p < 0.01 ***p < 0.001.

To determine whether TLR stimulation affects ACE2 and TMPRSS2 expression in HNECs, we examined mRNA expression in HNECs after stimulation with several TLR agonists for 24 hours. ACE2 mRNA expression was significantly increased upon TLR3-stimulation with Poly(I:C) (a 36.212±11.600-fold change, p<0.0001, **Figure 1A**). TMPRSS2 mRNA expression was also significantly increased upon TLR3-stimulation with Poly(I:C) (a 5.598±2.434-fold change, p=0.003, **Figure 1B**). These findings indicate that TLR3 stimulation with Poly(I:C) increases the expression levels of ACE2 and TMPRSS2.

### Poly(I:C) Upregulates Expression of NFκB Target Genes and an IFN-Stimulated Gene

We also examined the mRNA expression of TNF-α and IL-6 (NFκB target genes), as well as IFN-β, IFN-*γ*, and CXCL10 (an IFN-stimulated gene) in HNECs (**Supplementary Figure 1**). Poly(I:C) significantly promoted TNF-α (a 101.65±26.85-fold change, p<.0001) and IL-6 (a 215.85±34.82-fold change, p<.0001, **Supplementary Figures 1A, B**). Other than Poly(I:C), Pam3CSK4 (TLR1/2 agonist) significantly upregulated TNF-α (a 5.97±0.70-fold change, p=0.0046) and IL-6 (a 4.43±0.83-fold change, p=0.0233). FSL-1 (TLR6/2 agonist) also significantly upregulated TNF-α (a 7.68±2.56-fold change, p=0.002) and IL-6 (a 5.35±1.80-fold change, p=0.0132). Flagellin (a TLR5 agonist) and ODN2006 (a TLR9 agonist) significantly increased TNF-α (Flagellin; a 7.048±3.18-fold change, p=0.0051, ODN2006; a 5.15±1.92-fold change, p=0.0180).

As for the genes related to the IFN signaling pathway, Poly(I:C) also significantly upregulated the expression of IFN-β (a 14.19±4.37-fold change, p=0.0065) and CXCL10 (a 7496.34±1862.24-fold change, p<.0001). On the contrary, neither Pam3CSK4 nor FSL1 increased IFN-β or CXCL10 (**Supplementary Figures 1C, D**). IFN-*γ* was not upregulated by any TLR agonist (**Supplementary Figure 1E**). These results suggested that TLR3 stimulation with Poly(I:C) activates both the NFκB and IFN signaling pathways in HNECs.

### Poly(I:C) Induces ACE2 and TMPRSS2 mRNA Expression in a Time-Dependent Manner

**Figure 2:**
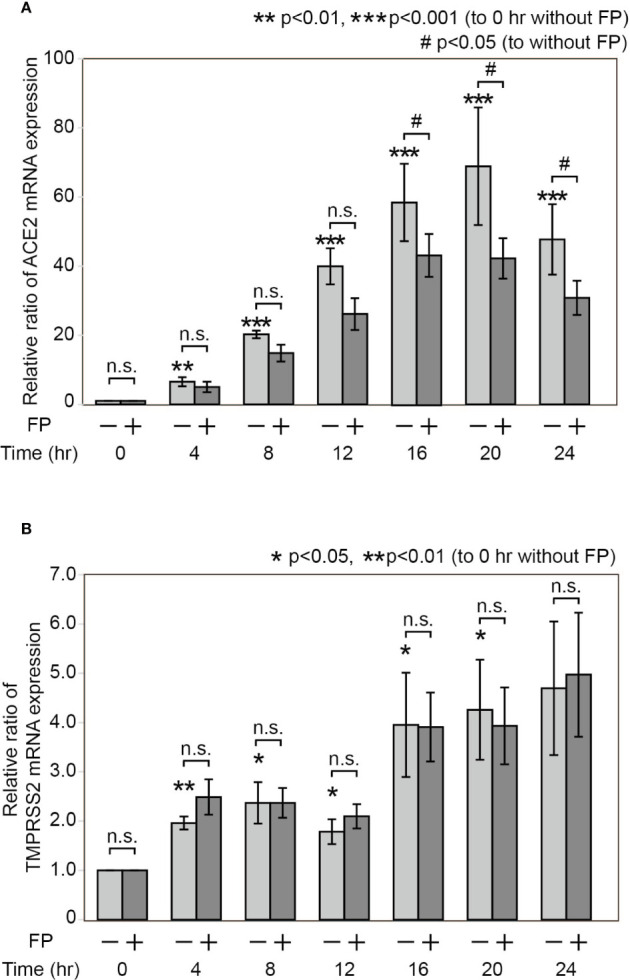
Time course of ACE2 and TMPRSS2 mRNA expression after Poly(I:C) stimulation with and without fluticasone propionate. HNECs were incubated for the indicated times with or without 10 µg/ml Poly(I:C) and 10 nM fluticasone propionate (FP), followed by qPCR using primer sets for ACE2 **(A)** and TMPRSS2 **(B)**. Data are means ± standard deviation (s.d.) of values from five independent experiments. P values for the indicated comparisons were determined by t-test with (ACE2) and without log transformation (TMPRSS2). *p < 0.05, **p < 0.01, ***p < 0.001 (for comparison between untreated cells and cells treated with Poly(I:C)), and ^#^p < 0.05 (for comparison between cells treated with and without FP at the indicated times, respectively).

We determined the time course of ACE2 mRNA expression after Poly(I:C) stimulation. A significant increase in ACE2 mRNA expression was found at 4, 8, 12, 16, 20 and 24 hours after stimulation with Poly(I:C) (**Figure 2A**). Similarly, a significant increase in TMPRSS2 mRNA expression was found at 4, 8, 12, 16, and 20 hours after stimulation with Poly(I:C) (**Figure 2B**).

We also examined ACE2 and TMPRSS2 mRNA expression in HNECs incubated with different concentrations of Poly(I:C). A significant increase in ACE2 mRNA expression was found after stimulation with 0.1, 1 and 10 µg/ml Poly(I:C) in a dose-dependency manner (**Supplementary Figure 2A**). On the other hand, a significant increase in TMPRSS2 mRNA expression was only observed after stimulation with 10 µg/ml Poly(I:C) (**Supplementary Figure 2B**).

### The Poly(I:C)-Induced Increase in ACE2 mRNA Expression Is Significantly Suppressed by FP

We investigated how FP affects ACE2 and TMPRSS2 expression in HNECs. A tendency for ACE2 expression to be suppressed by FP was seen at the indicated time points, and significant suppression of Poly(I:C)-induced ACE2 expression was observed at 16, 20, and 24 hours (**Figure 2A**). To the contrary, no suppression of TMPRSS2 mRNA expression by FP was observed at the indicated time points (**Figure 2B**).

We also examined the inhibitive effect on the Poly(I:C)-induced increases in ACE2 and TMPRSS2 mRNA expression of different concentrations of FP. Significant suppression of the Poly(I:C)-induced increases in ACE2 expression was observed for 1 and 10 nM FP (**Supplementary Figure 3A**). To the contrary, no suppression of TMPRSS2 mRNA expression was observed for any concentration of FP (**Supplementary Figure 3B**).

We also examined how FP affects the mRNA expression of genes related to the NFκB and IFN signaling pathways in HNECs. FP significantly suppressed the Poly(I:C)-induced increases in TNF-α and IL-6 expression as well as that of ACE2, while FP did not alter the Poly(I:C)-induced increases in IFN-β or CXCL10 expression (**Supplementary Figure 4**). Further, JSH-23, a NFκB transcriptional activity inhibitor, significantly suppressed the Poly(I:C)-induced increases in ACE2 (**Supplementary Figure 5A**) as well as those of the other NFκB target genes, TNF-α and IL-6, in HNECs (**Supplementary Figures 5B, C**), while JSH-23 did not alter the Poly(I:C)-induced increases in the expression of TMPRSS2 or the IFN-stimulated genes, IFN-β or CXCL10 (**Supplementary Figures 5D–F**). These results suggested that the Poly(I:C)-induced increases in ACE2 mRNA expression is induced *via* the NFκB signaling pathway, but not the IFN signaling pathway.

### Poly(I:C) and FP Significantly Affect ACE2, but Not TMPRSS2, Expression at the Protein Level

**Figure 3:**
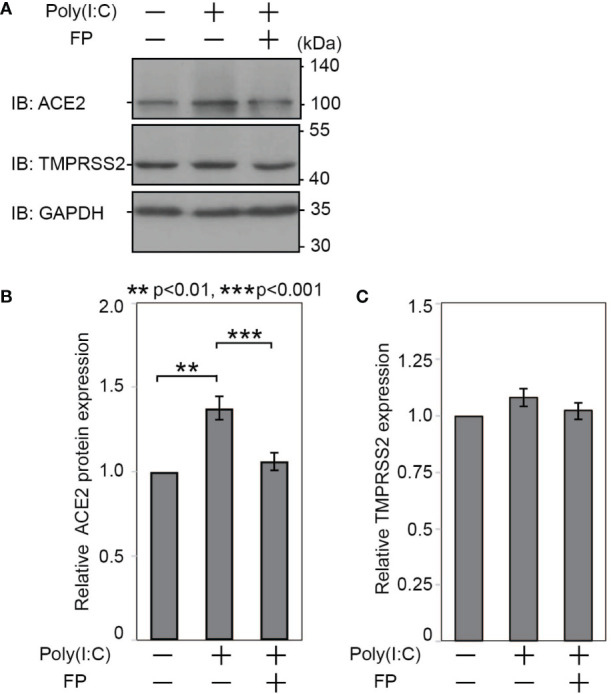
Immunoblotting for ACE2 and TMPRSS2 proteins in HNECs treated with Poly(I:C) and/or fluticasone propionate. HNECs were incubated for 28 hours with or without 10 µg/ml Poly(I:C) and 10 nM fluticasone propionate (FP). After removing oligosaccharides by PNGase F, the cell lysates were subjected to immunoblotting for ACE2 **(A, B)**, TMPRSS2 **(A, C)** and GAPDH. The obtained bands were quantified and calculated as relative ratios to GAPDH, using ImageJ 1.50i (National Institutes of Health, Bethesda, MD, USA). Data are means ± standard deviation (s.d.) of values from four independent experiments. P values for indicated comparisons were determined by t-test. **p < 0.01, ***p < 0.001.

Last, we examined whether Poly(I:C) and FP alter ACE2 and TMPRSS2 protein expression. Immunoblot analysis showed that Poly(I:C) significantly increased ACE2 protein expression (a 1.377±0.069-fold change *vs.* untreated cells, p=0.002). The Poly(I:C)-induced increase in ACE2 protein expression was significantly suppressed by FP (a 0.770±0.017-fold change *vs.* Poly(I:C)-treated cells, p<0.001, **Figures 3A, B**). On the other hand, there was no significant induction of TMPRSS2 protein expression by Poly(I:C) (a 1.083±0.0394-fold change *vs.* untreated cells, p=0.213) or subsequent suppression by FP (a 0.946±0.0340-fold change *vs.* Poly(I:C)-treated cells, p=0.208, **Figures 3A, C**).

**Figure 4:**
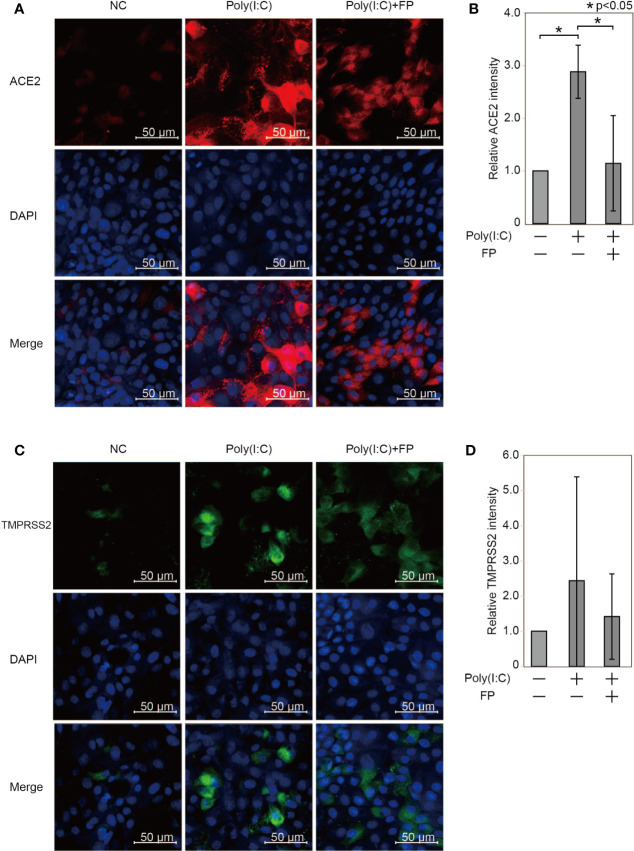
Immunofluorescent analysis for ACE2 and TMPRSS2 in HNECs treated with Poly(I:C) with and without fluticasone propionate. HNECs were incubated for 48 hours with or without 10 µg/ml Poly(I:C) and 10 nM fluticasone propionate (FP), followed by immunofluorescent analysis for ACE2 **(A, B)** and TMPRSS2 **(C, D)**. The average fluorescence intensity was standardized compared to the DAPI intensity and represented as the relative value to those of untreated cells. Data are means ± standard deviation (s.d.) of values from three independent experiments. P values for indicated comparisons were determined by t-test. *p < 0.05. The white bar is 50 µm and 20× magnification.

We further examined ACE2 and TMPRSS2 protein expression using immunofluorescent analysis (**Figure 4**). The ACE2 intensity in HNECs was significantly promoted by Poly(I:C) (a 2.884±0.505-fold change *vs.* untreated cells, p=0.003). This Poly(I:C)-induced change was significantly suppressed by FP (a 0.405±0.312-fold change *vs.* Poly(I:C)-treated cells, p=0.044, **Figures 4A, B**). The TMPRSS2 intensity was also altered by Poly(I:C) and FP, but the changes were not statistically significant (a 2.423±2.937-fold change *vs.* untreated cells p=0.449 and a 0.795±0.472-fold change *vs.* Poly(I:C)-treated cells, p=0.611, **Figures 4C, D**).

## Discussion

In this study, we demonstrated that innate immune signaling activation, in particular TLR3 activation, significantly promotes ACE2 expression in nasal epithelial cells. FP, a routinely used intranasal corticosteroid, suppressed the Poly(I:C)-induced increase in ACE2 expression but did not affect TMPRSS2 expression.

Understanding the regulation of SARS-CoV2 cell entry-related gene expression is important as it can directly lead to the study and development of preventive and therapeutic strategies against COVID-19 (Wu et al., 2020). To date, the focus has been mainly on ACE2 expression in the lower airway, rather than in the upper airway. In the lower airway, Poly(I:C), IFN-α, β, and *γ*, TNF-α, IL-6, and IL-1α and β were reported to activate the promoter of ACE2 (Zhuang et al., 2020; Ziegler et al., 2020). Also, viral infection and smoking promote ACE2 expression (Kimura et al., 2020; Sharif-Askari et al., 2020; Zhuang et al., 2020). Conversely, type 2 inflammatory cytokines have been shown to suppress ACE2 expression (Jackson et al., 2020; Kimura et al., 2020). Notably, this is thought to potentially contribute to a decreased risk of COVID-19 in patients with asthma (Matsumoto and Saito, 2020).

On the other hand, little is known about ACE2 expression in the upper airway. Significant differences in the innate immune response have been reported between nasal and bronchial epithelial cells (Cooksley et al., 2015). Hence, findings specific to the lower airway cannot automatically be generalized to apply to the upper airway immune response without further detailed studies. To date, only IFN-*γ* and type 2 inflammatory cytokines have been reported to affect ACE2 expression in the upper airway (Kimura et al., 2020; Ziegler et al., 2020). As for TMPRSS2 expression, current knowledge is limited for both the upper and lower airway, with only the androgen receptor reported to upregulate TMPRSS2 expression, although this finding has not been confirmed in the airway (Lin et al., 1999; Lucas et al., 2014). Whether TMPRSS2 expression is regulated by the activated innate immune signals in the nasal mucosa has not yet been elucidated.

In this study, we found that ACE2 expression in HNECs was significantly increased by stimulation with Poly(I:C), as many other genes in HNECs were found to do in previous reports, including IL-6 (Cooksley et al., 2015), MMP9, MMP1, MMP10, TIMP-1 (Suzuki et al., 2018), TSLP (Golebski et al., 2016), TNF-α and IL-8 (Ohkuni et al., 2011). Poly(I:C) is a double-stranded RNA (dsRNA) often used as a model for viral infection, as most viruses induce the synthesis of dsRNA during their replication cycle (Jacobs and Langland, 1996). This finding might afford an important insight into the prevention and treatment of acute rhinosinusitis by viral infection, not only in terms of relieving its nasal symptoms but also in preventing secondary SARS-CoV-2 infection. Further research is required to evaluate the potential role of increased ACE2 expression in enhancing SARS-CoV-2 infection.

Another highlight of this study was the suppressive effect of FP on Poly(I:C)-induced increases in ACE2 expression in nasal epithelial cells. From a clinical view point, controversy remains surrounding the use of corticosteroids for COVID-19 (Russell et al., 2020; Shang et al., 2020). Although clinical evidence does not support systemic corticosteroid treatment for COVID-19 (Russell et al., 2020), ciclesonide, a widely-used inhalant corticosteroid, could be beneficial against COVID-19 in preventing SARS-CoV-2 replication (Matsuyama et al., 2020). As for intra-nasal corticosteroids, The European Academy of Allergy and Clinical Immunology (EAACI) has stated that intra-nasal corticosteroids can be continued for cases of allergic rhinitis and that halting their use is not advised as no immunosuppression has been shown after intranasal corticosteroid use and the increase in sneezing after halting their application may lead to a greater spread of SARS-CoV-2 (Bousquet et al., 2020). However, intranasal corticosteroids might impair anti-viral immune responses and increase viral titers, as they do for rhinovirus (Singanayagam et al., 2015). Further studies are needed to clarify the clinical significance of intranasal steroid spray for COVID-19.

**Figure 5:**
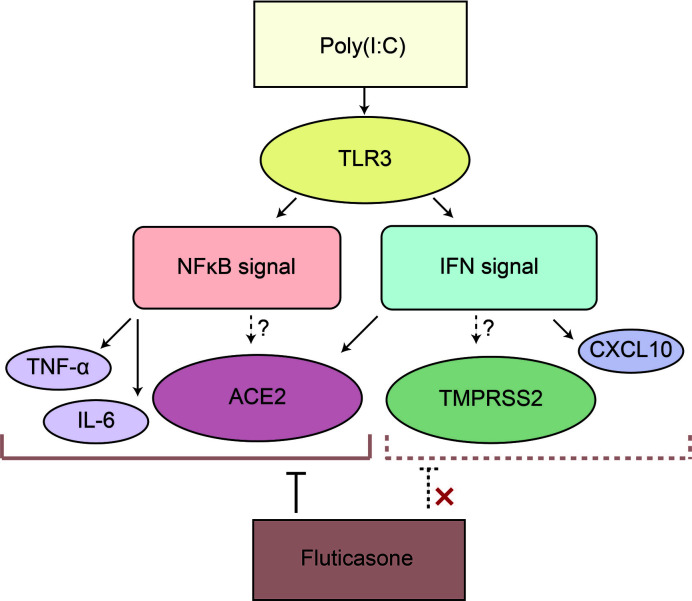
Poly(I:C) significantly increased ACE2 and TMPRSS2 possibly via the IFN and NFκB signaling pathways in HNECs. Poly(I:C) activates both the IFN and NFκB signaling pathways by binding to TLR3, resulting in the induction of ACE2 and TMPRSS2. IFN-β upregulates ACE2 expression; however, ACE2 as well as the target genes of the NFκB signaling pathway such as TNF-α and IL-6 is suppressed by fluticasone propionate, while TMPRSS2 and an interferon-stimulated gene are not. This suggested that ACE2 in HNECs is regulated not only *via* the IFN signaling pathway but also *via* the NFκB signaling pathway.

Interestingly, we found different responses to FP in the NFκB and IFN signaling pathways. While FP suppressed the NFκB target genes, FP did not suppress IFN-β itself or IFN-stimulated genes in HNECs. Glucocorticoids including FP induced gene transcription and protein synthesis of the NFκB inhibitor, IκB. Activated glucocorticoid cytosolic receptors also antagonized NFκB activity through protein–protein interaction involving direct complexing with, and inhibition of, NFκB binding to DNA (Almawi and Melemedjian, 2002). On the other hand, it is reported that the response to the IFN signaling pathway by glucocorticoids varies depending on cell type (Flammer et al., 2010). In HNECs, the Poly(I:C)-induced increase in ACE2 was also suppressed by FP, suggesting that ACE2 is significantly regulated *via* the NFκB signaling pathway, as well (Hou et al., 2020). To the contrary, the Poly(I:C)-induced increase in TMPRSS2 was not suppressed by FP, implying that the NFκB signaling pathway plays a limited role in the regulation of TMPRSS2 expression (**Figure 5**). Furthermore, we found that a NFκB inhibitor significantly suppressed the Poly(I:C)-induced increase in ACE2 expression, suggesting this increase is regulated *via* the NFκB signaling pathway in nasal epithelial cells.

As a limitation, although we demonstrated that activated innate immune signals promoted the expression of SARS-CoV2 cell entry-related genes on which SARS-CoV-2 cell entry is dependent the association between the upregulated expression of the genes and host susceptibility to SARS-CoV-2 remains to be investigated. Also, it is unknown whether the suppression of ACE2 expression by FP can indeed prevent SARS-CoV-2 infection. Although further biological and epidemiological studies are necessary, it can be concluded that the regulation of inflammation in the nasal cavity is increasingly important and should be of greater focus in the COVID-19 era than ever before.

## Conclusion

The activation of innate immune signals *via* TLR3 promotes the expression of SARS-CoV2 cell entry-related genes in the nasal mucosa, which is suppressed in the presence of the intranasal corticosteroid FP. Further studies are required to evaluate whether FP suppresses SARS-CoV-2 viral cell entry.

## Data Availability Statement

The original contributions presented in the study are included in the article/**Supplementary Material**, further inquiries can be directed to the corresponding author.

## Ethics Statement

This study was approved by the Institutional Review Board for Clinical Research of Hokkaido University Hospital, Sapporo, Japan (019–0242) and by The Queen Elizabeth Hospital Human Research Ethics Committee (reference HREC/15/TQEH/132). The patients/participants provided their written informed consent to participate in this study.

## Author Contributions

AN, YN, and MS designed the experiments. SH, PW, SV, and AkH supervised the project. AN, MR, MW, and TK performed most of the experiments. AN, SK, AyH, and MS compiled the data. AN, SV, SH and MS wrote the manuscript. All authors contributed to the article and approved the submitted version.

## Funding

This study was supported by JSPS KAKENHI Grant Number 19K18718 for AN, 17H06491, 18K16871, and 18KK0444 for MS, 20K18301 for AyH and 20K09703 for YN, GSK Japan Research Grant 2015, Akiyama Life Science Foundation grants to MS and AyH and a Sanofi Japan research grant to AkH.

## Conflict of Interest

The authors declare that the research was conducted in the absence of any commercial or financial relationships that could be construed as a potential conflict of interest.

## Supplementary Material

Supplementary Figure 1 Poly(I:C) significantly increases the expression of NFκB target genes and genes related to the IFN signaling pathway in HNECs. Relative mRNA expression of TNF-α **(A)**, IL-6 **(B)**, IFN-β **(C)**, CXCL10 **(D)**, and IFN-*γ* (E) after stimulation with TLR agonists for 24 hours. The following TLR agonists were used: Pam3CSK4 for TLR1/2, HKLM for TLR2, Poly(I:C) for TLR3, LPS for TLR4, Flagellin for TLR5, FSL-1 for TLR6/2, Imiquimod for TLR7, ssRNA40 for TLR8, and ODN2006 for TLR9. Relative mRNA expression was determined by normalization against untreated control cells and GAPDH. Data are means ± standard deviation (s.d.) of values from five independent experiments. *p<0.05 **p<0.01 ***p<0.001.

Supplementary Figure 2 ACE2 and TMPRSS2 mRNA expression in HNECs incubated with different concentrations of Poly(I:C). Relative mRNA expression of ACE2 **(A)** and TMPRSS2 **(B)** after stimulation with 0.1, 1 and 10 µg/ml Poly(I:C) for 20 hours. Relative mRNA expression was determined by normalization against untreated control cells and GAPDH. Data are means ± standard deviation (s.d.) of values from five independent experiments. *p<0.05 ***p<0.001.

Supplementary Figure 3 ACE2 and TMPRSS2 mRNA expression in HNECs incubated with different concentrations of fluticasone propionate. HNECs were incubated for 20 hours with 10 µg/ml Poly(I:C) and/or 0.1, 1 and 10 nM fluticasone propionate (FP), followed by qPCR using primer sets for ACE2 **(A)** and TMPRSS2 **(B)**. Relative mRNA expression was determined by normalization against untreated control cells and GAPDH. Data are means ± standard deviation (s.d.) of values from four independent experiments. *p<0.05.

Supplementary Figure 4 Fluticasone propionate significantly suppressed the Poly(I:C)-induced increase in the expression of NFκB target genes but not genes related to the IFN signaling pathway. HNECs were incubated for 8 hours with 10 µg/ml Poly(I:C) and/or 10 nM fluticasone propionate (FP), followed by qPCR using primer sets for TNF-α **(A)**, IL-6 **(B)**, IFN-β **(C)** and CXCL10 **(D)**. Data are means ± standard deviation (s.d.) of values from three or four independent experiments. P values for indicated comparisons were determined by t-test with log transformation. *p<0.05, **p<0.01, ***p<0.001.

Supplementary Figure 5 A NFκB inhibitor significantly suppressed ACE2 expression in HNECs. Relative mRNA expression of ACE2 **(A)**, TNF-α **(B)**, IL-6 **(C)**, TMPRSS2 **(D)**, IFN-β **(E)** and CXCL10 **(F)** in HNECs incubated with 10 µg/ml Poly(I:C) and 30 µM JSH-23 (NFκB transcriptional activity inhibitor) for 20 hours (for ACE2 and TMPRSS2) and for 8 hours (for TNF-α, IL-6, IFN-β, and CXCL10), respectively. Relative mRNA expression was determined by normalization against untreated control cells and GAPDH. Data are means ± standard deviation (s.d.) of values from four independent experiments. *p<0.05 **p<0.01 ***p<0.001.
