# Supplementary figures and images for: Fluticasone Propionate Suppresses Poly(I:C)-Induced ACE2 in Primary Human Nasal Epithelial Cells
Source: Front Cell Infect Microbiol. 2021 Apr 26;11:655666. doi: 10.3389/fcimb.2021.655666 (PMC8107375; doi:10.3389/fcimb.2021.655666)

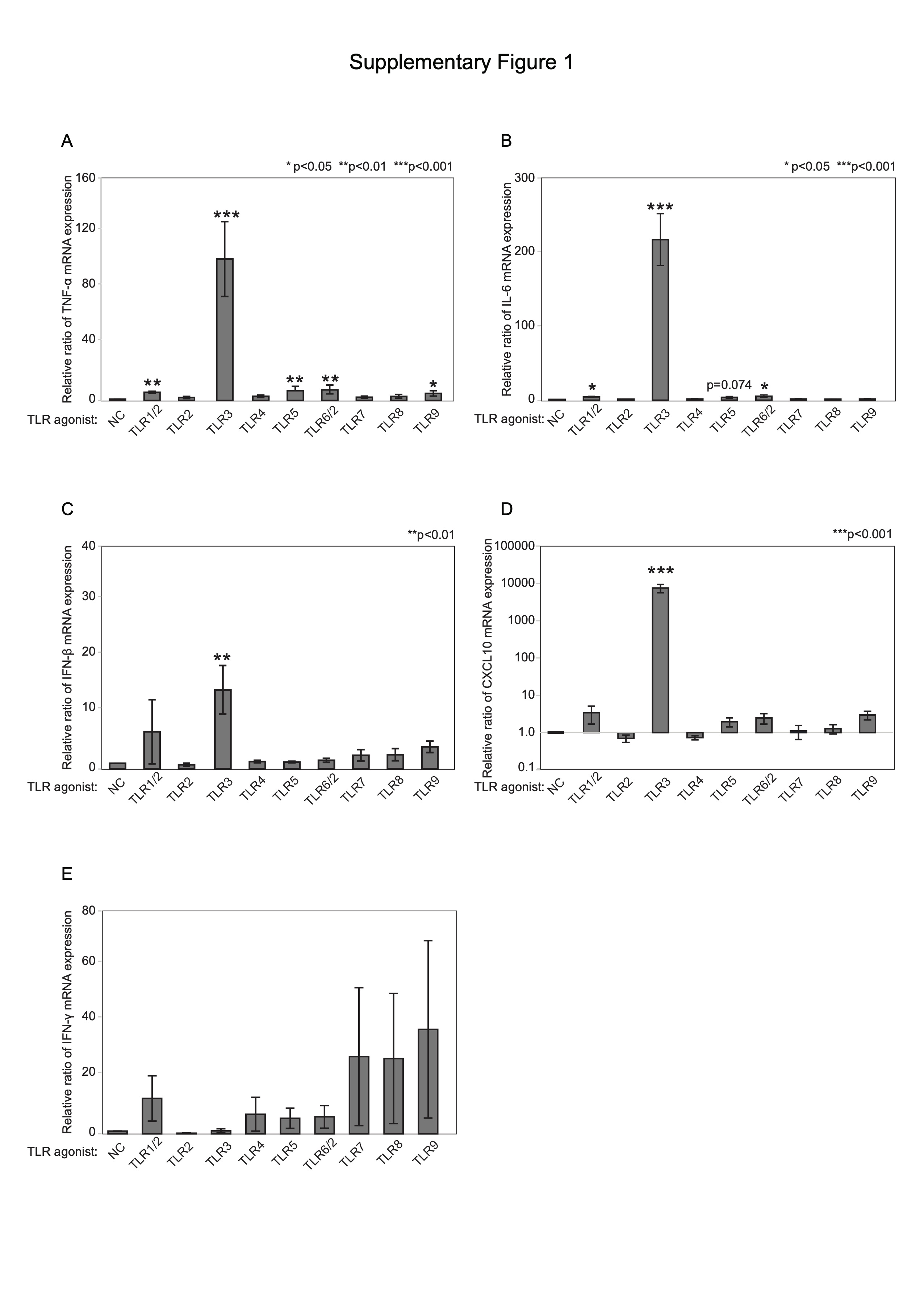

Supplement: Supplementary Figure 1 — Poly(I:C) significantly increases the expression of NFκB target genes and genes related to the IFN signaling pathway in HNECs. Relative mRNA expression of TNF-α (A), IL-6 (B), IFN-β (C), CXCL10 (D), and IFN-γ (E) after stimulation with TLR agonists for 24 hours. The following TLR agonists were used: Pam3CSK4 for TLR1/2, HKLM for TLR2, Poly(I:C) for TLR3, LPS for TLR4, Flagellin for TLR5, FSL-1 for TLR6/2, Imiquimod for TLR7, ssRNA40 for TLR8, and ODN2006 for TLR9. Relative mRNA expression was determined by normalization against untreated control cells and GAPDH. Data are means ± standard deviation (s.d.) of values from five independent experiments. *p<0.05 **p<0.01 ***p<0.001. [file Image_1.jpg]

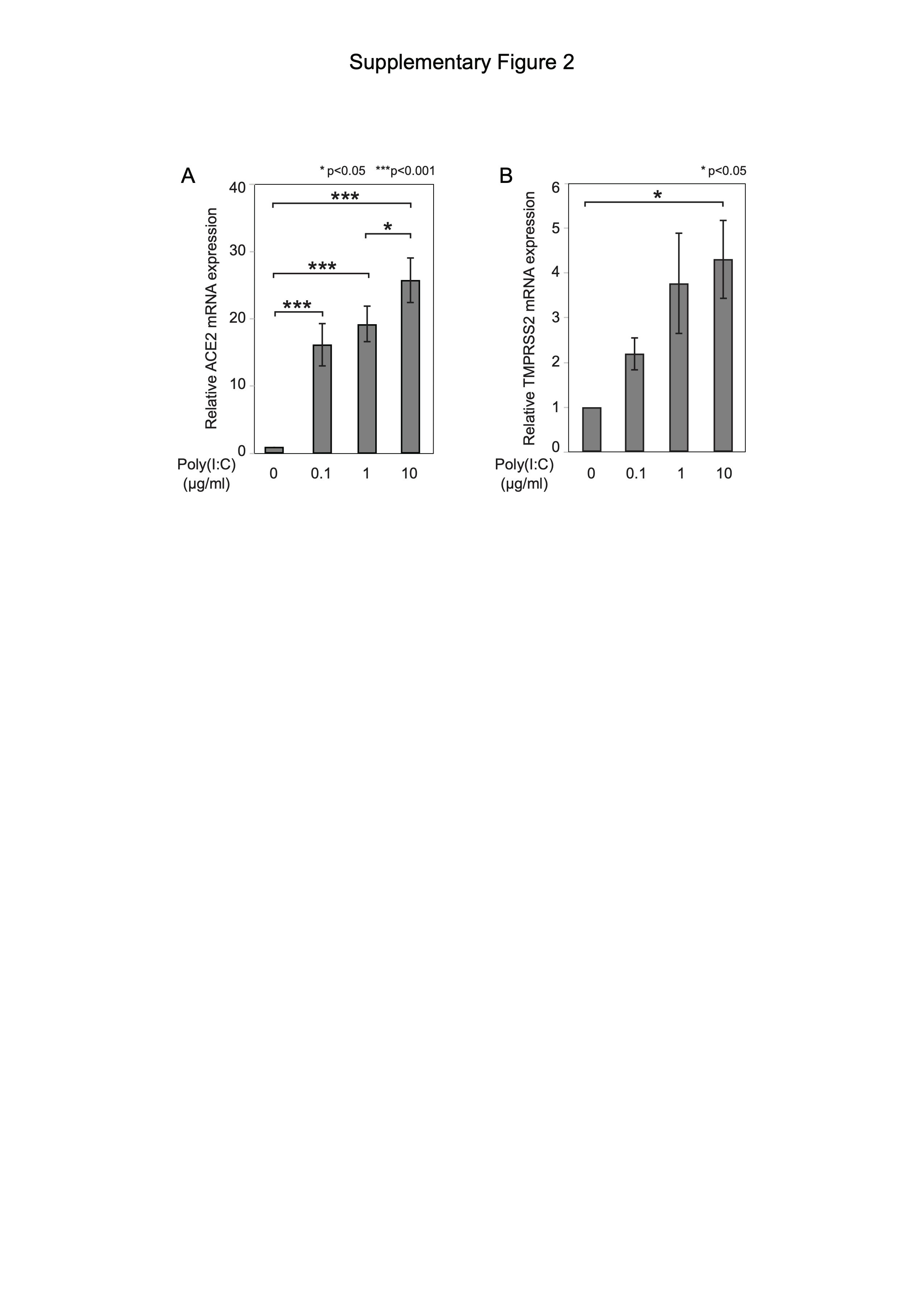

Supplement: Supplementary Figure 2 — ACE2 and TMPRSS2 mRNA expression in HNECs incubated with different concentrations of Poly(I:C). Relative mRNA expression of ACE2 (A) and TMPRSS2 (B) after stimulation with 0.1, 1 and 10 µg/ml Poly(I:C) for 20 hours. Relative mRNA expression was determined by normalization against untreated control cells and GAPDH. Data are means ± standard deviation (s.d.) of values from five independent experiments. *p<0.05 ***p<0.001. [file Image_2.jpg]

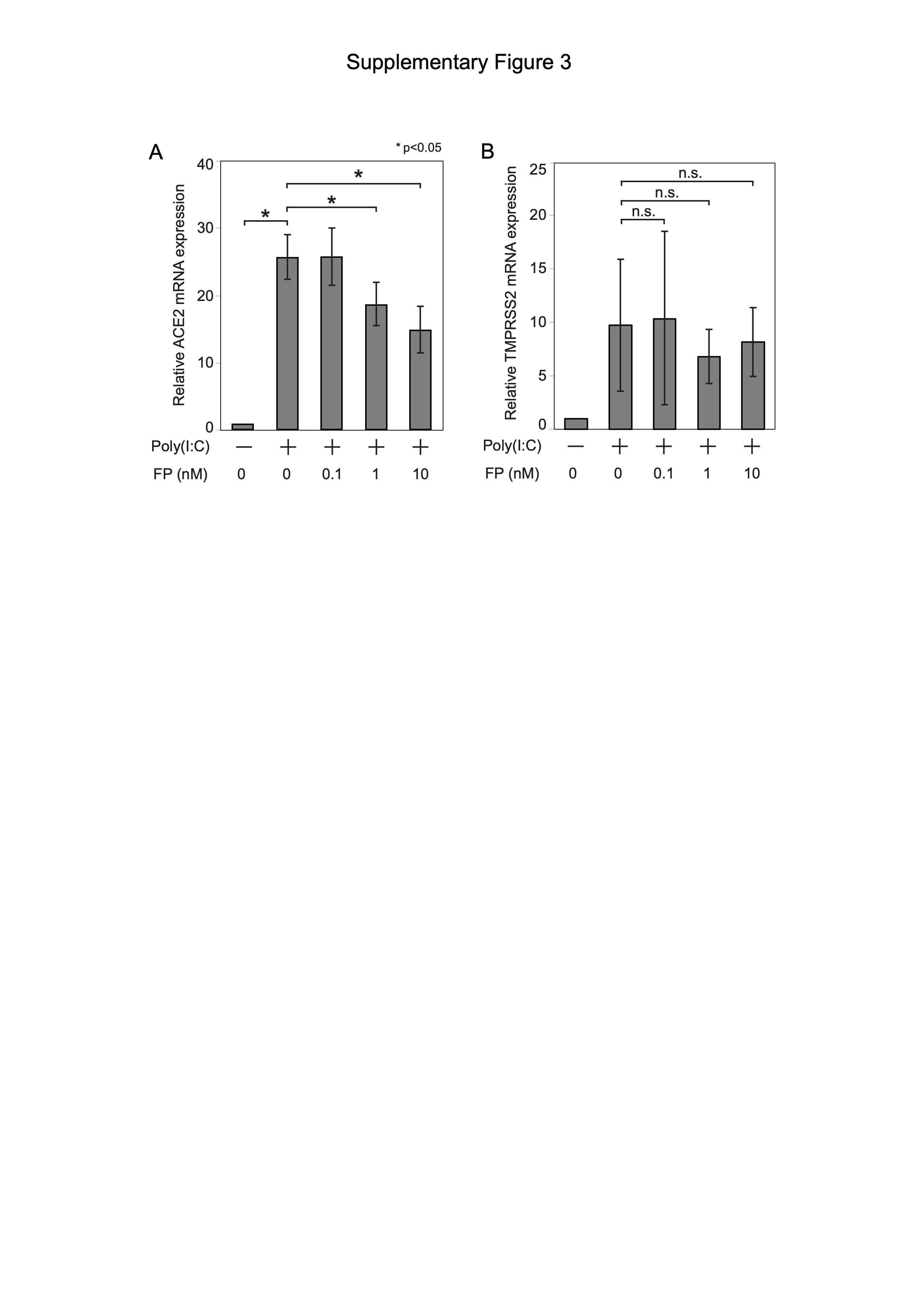

Supplement: Supplementary Figure 3 — ACE2 and TMPRSS2 mRNA expression in HNECs incubated with different concentrations of fluticasone propionate. HNECs were incubated for 20 hours with 10 µg/ml Poly(I:C) and/or 0.1, 1 and 10 nM fluticasone propionate (FP), followed by qPCR using primer sets for ACE2 (A) and TMPRSS2 (B). Relative mRNA expression was determined by normalization against untreated control cells and GAPDH. Data are means ± standard deviation (s.d.) of values from four independent experiments. *p<0.05. [file Image_3.jpg]

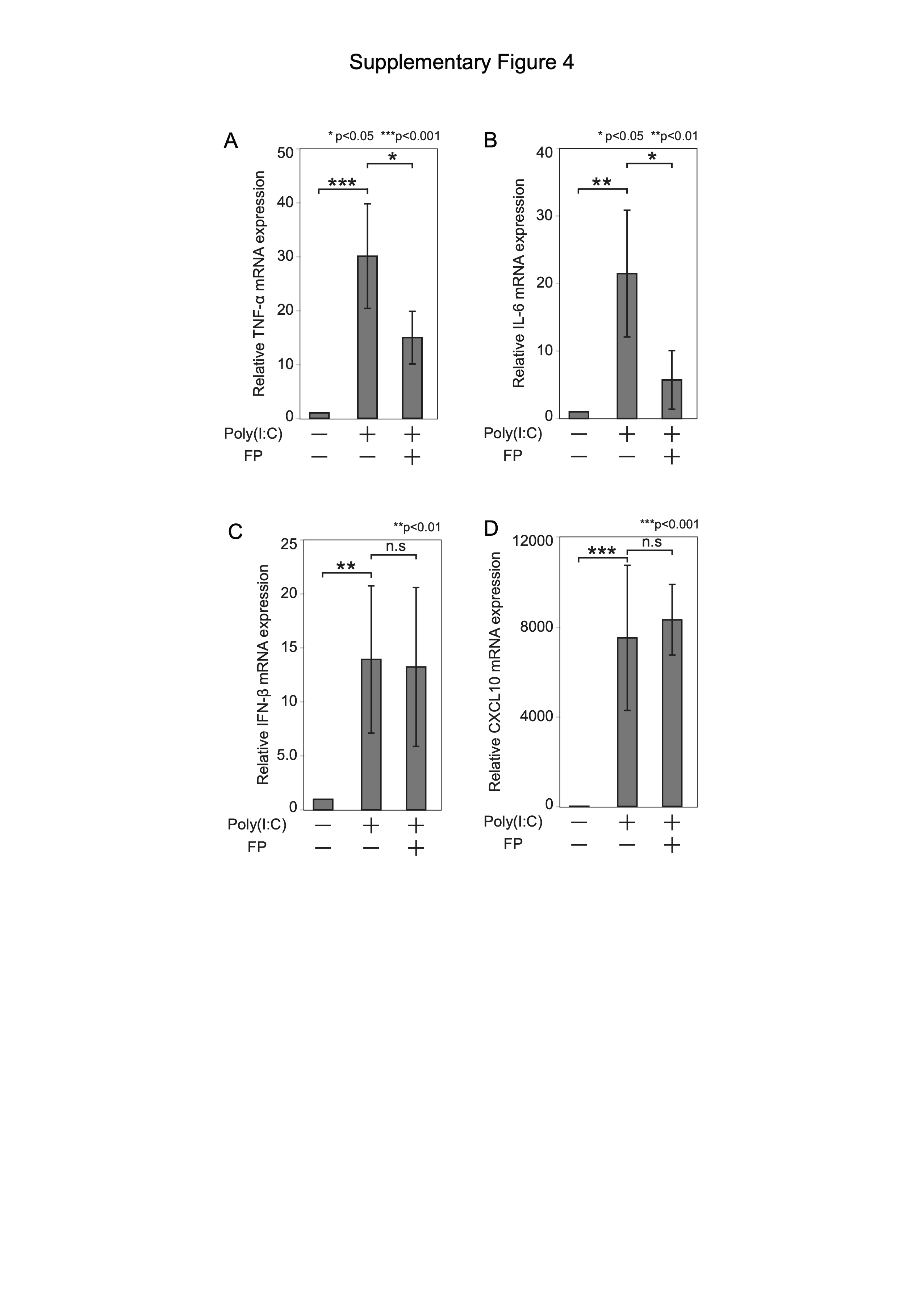

Supplement: Supplementary Figure 4 — Fluticasone propionate significantly suppressed the Poly(I:C)-induced increase in the expression of NFκB target genes but not genes related to the IFN signaling pathway. HNECs were incubated for 8 hours with 10 µg/ml Poly(I:C) and/or 10 nM fluticasone propionate (FP), followed by qPCR using primer sets for TNF-α (A), IL-6 (B), IFN-β (C) and CXCL10 (D). Data are means ± standard deviation (s.d.) of values from three or four independent experiments. P values for indicated comparisons were determined by t-test with log transformation. *p<0.05, **p<0.01, ***p<0.001. [file Image_4.jpg]

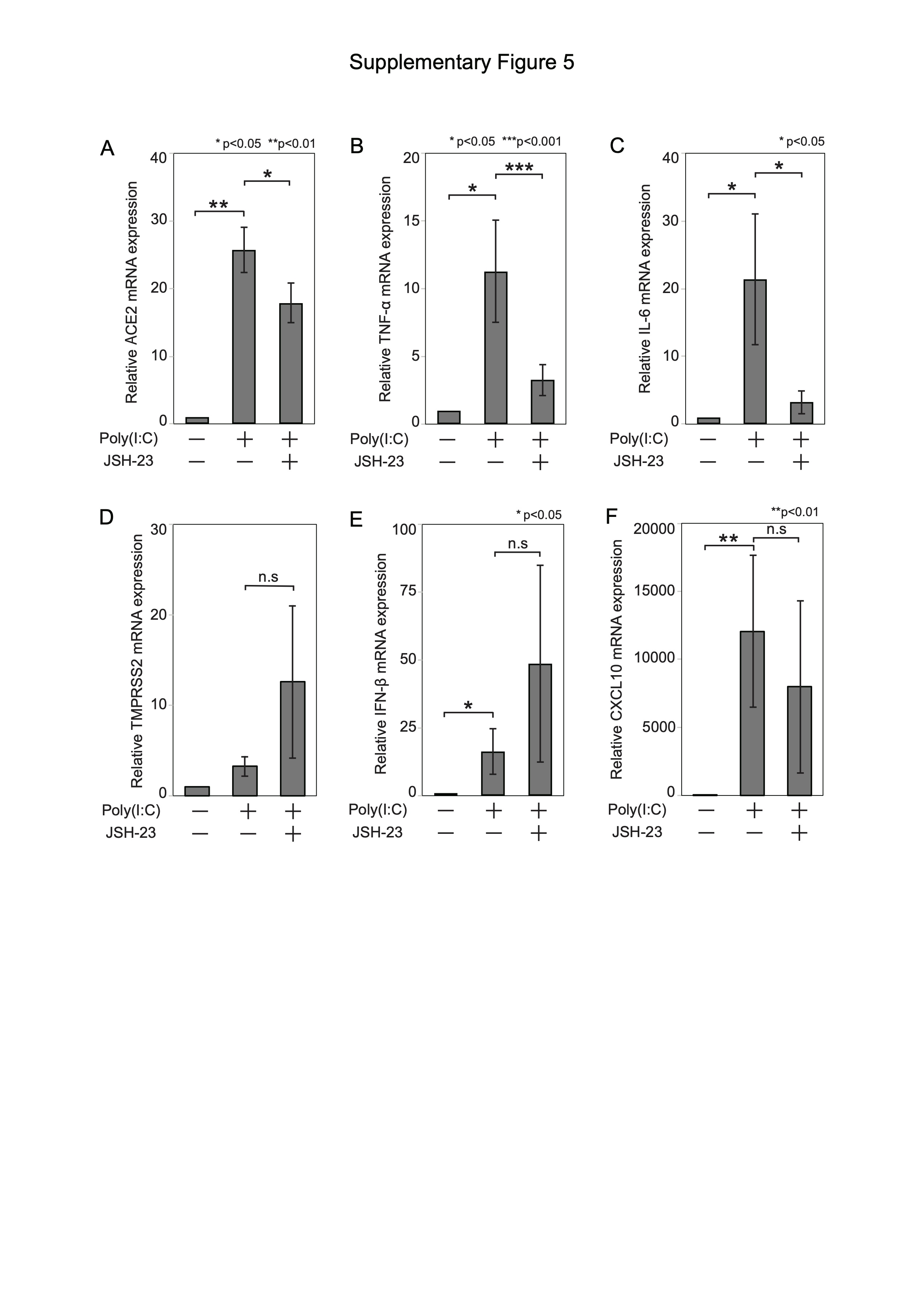

Supplement: Supplementary Figure 5 — A NFκB inhibitor significantly suppressed ACE2 expression in HNECs. Relative mRNA expression of ACE2 (A), TNF-α (B), IL-6 (C), TMPRSS2 (D), IFN-β (E) and CXCL10 (F) in HNECs incubated with 10 µg/ml Poly(I:C) and 30 µM JSH-23 (NFκB transcriptional activity inhibitor) for 20 hours (for ACE2 and TMPRSS2) and for 8 hours (for TNF-α, IL-6, IFN-β, and CXCL10), respectively. Relative mRNA expression was determined by normalization against untreated control cells and GAPDH. Data are means ± standard deviation (s.d.) of values from four independent experiments. *p<0.05 **p<0.01 ***p<0.001. [file Image_5.jpg]
